# Volta phase plate cryo-EM of the small protein complex Prx3

**DOI:** 10.1038/ncomms10534

**Published:** 2016-01-28

**Authors:** Maryam Khoshouei, Mazdak Radjainia, Amy J. Phillips, Juliet A. Gerrard, Alok K. Mitra, Jürgen M. Plitzko, Wolfgang Baumeister, Radostin Danev

**Affiliations:** 1 Department of Molecular Structural Biology, Max Planck Institute of Biochemistry, 82152 Martinsried, Germany; 2 School of Biological Sciences, University of Auckland, Auckland 1042, New Zealand; 3 Department of Biochemistry and Molecular Biology, Monash University, Victoria, 3800 Melbourne, Australia; 4 Lincoln Agritech Limited, Lincoln 7640, New Zealand; 5 MacDiarmid Institute for Advanced Materials and Nanotechnology, Victoria University, Wellington 6140, New Zealand; 6 School of Chemical Sciences, University of Auckland, 23 Symonds Street, Auckland 1010, New Zealand

## Abstract

Cryo-EM of large, macromolecular assemblies has seen a significant increase in the numbers of high-resolution structures since the arrival of direct electron detectors. However, sub-nanometre resolution cryo-EM structures are rare compared with crystal structure depositions, particularly for relatively small particles (<400 kDa). Here we demonstrate the benefits of Volta phase plates for single-particle analysis by time-efficient cryo-EM structure determination of 257 kDa human peroxiredoxin-3 dodecamers at 4.4 Å resolution. The Volta phase plate improves the applicability of cryo-EM for small molecules and accelerates structure determination.

## Abstract (web summary)

Although the resolution achievable with cryo-EM can now rival crystallography, its application to small protein assemblies remains challenging. Here the authors demonstrate the use of the Volta phase plate for single particle analysis and its potential for the study of small specimens.

## Graphical abstract

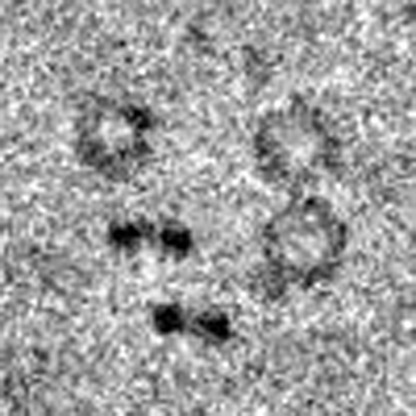

Cryo-electron microscopy (cryo-EM) has long held the promise to deliver high-resolution structures of biological assemblies without the need for crystallization^1^. Direct electron detectors are proving pivotal in fulfilling this promise, with the recent publications of many atomic or near-atomic models derived from cryo-EM maps^2^. However, successes such as those reported by Liao *et al*.^3^, Bai *et al*.^4^ and Bartesaghi *et al*.^5^ often represent well-behaved specimens that are exceptions rather than the norm. The challenges in obtaining successful, high-resolution cryo-EM images are manifold. Factors that ultimately dictate the attainable resolution include particle size, rigidity, sample purity, orientation coverage and ice thickness^6^. Optimizing these parameters can be complex, prohibitively time-consuming, and does not always lead to high-quality maps. These potential issues severely limit a broader applicability for single-particle cryo-EM.

The major impediment in achieving sub-nanometre resolution cryo-EM reconstructions across the proteome is the intrinsically low signal-to-noise ratio obtainable with such unstained biological samples embedded in ice^1^. The recently proposed Volta potential phase plate (VPP) addresses this long-recognized problem of cryo-EM^7^,^8^ and has many advantages over previous phase plate designs including durability and ease of use^9^. It is a remarkably simple device, comprising a heated film of continuous, amorphous carbon located at the back-focal plane of the objective lens (Supplementary Fig. 1). The VPP introduces a phase shift in the unscattered beam relative to scattered beams, increasing the phase contrast, and thus facilitates the observation of weak phase objects with markedly improved contrast.

While the use of VPP has already proven essential in cryo-electron tomography studies to reveal structural details of 26S proteasomes in intact neurons^10^, its benefits to single-particle analysis have not yet been demonstrated. We sought to investigate the potential for structure determination using VPP with single-particle analysis, specifically for solving the cryo-EM structure of the 257-kDa, toroidal dodecamers of human peroxiredoxin-3 (hPrx3; ref. 11) as an example for tackling relatively ‘small' protein complexes.

Peroxiredoxins (Prxs) are a ubiquitous class of peroxidases that mediate the detoxification of reactive oxygen species or detection of cellular stress, with a concomitant change of quaternary organization^12^. hPrx3 is a human mitochondrial isoform that forms dodecamers when the central cysteine is in its reduced form. The dodecamers can further assemble into long filaments upon acidification^13^. We recently obtained cryo-EM reconstructions of hPrx3 filaments and observed a structured C terminus^11^, which has not been previously solved in homologous crystal structures, such as that of bovine Prx3 (ref. 14).

Determination of high-resolution structures for non-crystalline hPrx3 samples can be laborious in practice as recently reviewed by Cheng *et al*.^6^. This is owing to the following two reasons: first, complexes with a molecular weight below 300 kDa exhibit very poor contrast and are particularly susceptible to beam-induced motion; and second, toroidal structures such as the hPrx3 dodecamers often assume preferred orientations in thin ice layers and at low concentrations^6^. These issues can be overcome by using thicker ice and increased protein concentration, but this leads to complications in terms of further reduced contrast and difficulties in particle detection^6^.

In this study, we report the 4.4-Å resolution structure of 257 kDa hPrx3, which paves the way for routinely determining relatively small complexes at near-atomic resolution by VPP-enabled phase contrast cryo-EM.

## Results

### 3D reconstruction from a manually collected dataset

We sought to elucidate the conformation that hPrx3 assumes in solution, without the potential for structural changes that may occur during assembly to higher-order structures or crystal packing. Frozen-hydrated specimens of hPrx3 were prepared by relaxing the blot force of the automatic plunging device to increase ice thickness. On the first attempt, hPrx3 particles were visualized with high contrast, dense distribution and random orientations (Supplementary Fig. 2). Forty-four in-focus images were recorded manually in movie mode, from which a 7.3-Å single-particle cryo-EM reconstruction was obtained, resolving secondary structure elements (Supplementary Fig. 3).

Already, at this level of resolution, α-helices are clearly discernible and reveal a volume of density that can be assigned to a folded C terminus, similar to that seen in the reconstructions of hPrx3 filaments as well as other Prx isoforms^15^ (Supplementary Fig. 3). This finding highlights the strengths of using VPP for single-particle analysis, which include greatly reduced requirements for optimization of sample preparation, and structure determination to sub-nanometre resolution with a relatively small number of images. Therefore, one can efficiently delineate the structure of a complex in solution to complement structural information obtained by X-ray crystallography. The ability to induce and visualize alternate orientations of hPrx3 using thicker ice is particularly noteworthy, which would have otherwise restricted us to a low-resolution random conical tilt reconstruction. In fact, we attempted to reconstruct hPrx3 without phase plates using a grid from the same batch that yielded the 7.3-Å phase plate structure. However, we were not able to obtain a reconstruction that is consistent with the structure of hPrx3, let alone well resolved, despite investing more effort in particle picking and processing. Both data sets were made available for readers that would like to compare the respective raw data.

### 3D reconstruction from an automatically collected dataset

**Figure 1:**
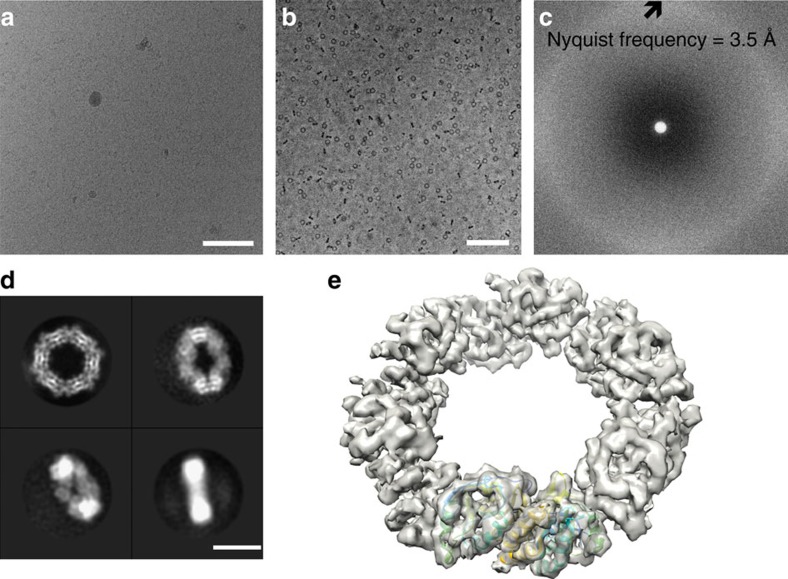
Near-atomic resolution reconstruction of hPrx3. (**a**) Electron micrograph of frozen-hydrated hPrx3 dodecamers taken without a phase plate at 2.4 μm underfocus. In thick ice, toroids are only readily discernible at high defocus (scale bar, 100 nm). (**b**) In-focus electron micrograph of hPrx3 particles. VPP images of hPrx3 are high in contrast even in-focus and before motion correction, enabling robust automated particle selection of all views (scale bar, 100 nm). (**c**) Power spectrum of electron micrograph featuring continuous signal without contrast transfer function (CTF) oscillations across the frequency spectrum. (**d**) Four representative class averages of hPrx3 featuring random orientations promoted by thick ice (scale bar, 10 nm). (**e**) Isosurface representation of the reconstructed 3D density map with bovine Prx3 dimer structure docked (PDB 1ZYE (ref. 14)).

**Figure 2:**
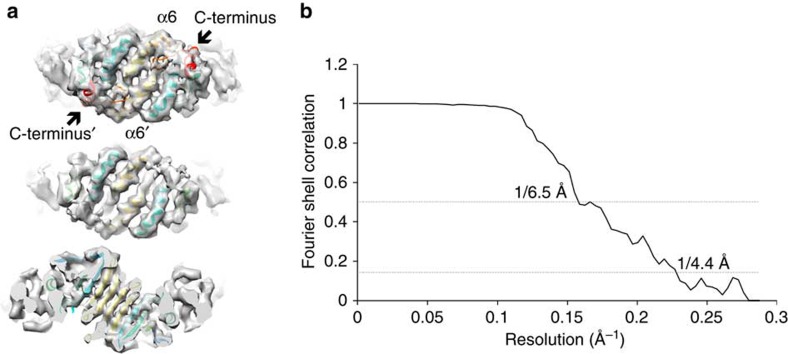
3D density map with docked X-ray structures. (**a**) The VPP reconstruction of hPrx3 is consistent with the crystal structure of hPrx2 (PDB 1QMV (ref. 15)), where the dimer features folded C terminus (red; top). The C-terminal region is not present in the crystal structure of the bovine homologue of Prx3 (PDB 1ZYE (ref. 14)) and disappears in our EM map at lower thresholds (middle), suggesting only partial occupancy of the folded C terminus. The obtained resolution is sufficient for resolving individual beta-strands in the density map (bottom). (**b**) Fourier-shell correlation indicates a resolution of 4.4 Å based on the ‘gold-standard' Fourier shell correlation (FSC)=0.143 criterion.

Since our first VPP data set of hPrx3 featured a significant amount of drift due to instability of the support film, we recorded another data set using a different grid type to see whether resolution can be improved. Indeed, with the new data set, we were able to obtain a reconstruction of hPrxs at a resolution of 4.4 Å from 8,500 particles (Fig. 1). This boost in resolution markedly improved the quality of the map allowing for more precise interpretation of secondary structure motifs, including the C-terminal alpha helix, which was not fully resolved in the 7.3-Å map (Fig. 2).

This result clearly shows that VPP imaging is not limited to intermediate resolutions, which has been observed for other phase plate designs^16^ and can, in the case of hPrx3, even deliver a near-atomic resolution. It should also be noted that the second map was sharpened with a smaller B-factor of −137. This value suggests a less steep fall-off of high-resolution information, indicating that even higher resolution reconstructions may be possible using VPP.

## Discussion

In close-to-focus images taken with a VPP, contrast transfer is maximal and continuous for low-to-high spatial frequencies. The principles underlying this phenomenon are similar to light microscopy where phase plates have been commonplace since the 1940s (ref. 17), and which have been finally realized in a practical solution for transmission electron microscopes through the VPP.

To have maximum contrast and obtain sub-8 Å resolution surpasses current alternatives for many applications^8^. Without phase plates, microscopists can primarily defocus the objective lens—a deliberate aberration of the microscope optics—to increase the contrast of weak phase objects. This is not an issue with ideal cryo-EM specimens, for which molecular weight and isotropic coverage in thin ice are not limiting factors. In many cases, however, a useful compromise between contrast and resolution cannot be obtained, particularly when contrast is extremely weak and very high defocus levels are needed to compensate^6^. Defocusing introduces oscillations in the transfer of contrast as a function of spatial frequency, which together with the effect of the envelope function attenuate high-resolution components in the image^6^.

Despite the ∼18% signal loss with the VPP^9^, it is still possible to obtain near-atomic resolution structures even for a relatively small complex such as hPrx3. This contrasts with previous efforts using alternative phase plate designs where signal decay at high resolution, reflected in very high B-factors, impedes high-resolution structure determination^16^.

We believe that VPP single-particle analysis will add great value in two ways. First, it can act in a complementary role to high-resolution structure determination techniques such as X-ray crystallography, defocus contrast-based single-particle analysis or nuclear magnetic resonance, and will be a powerful alternative to negative staining techniques or small-angle X-ray scattering. Second, the example of hPrx3 also heralds a role for phase plate technology as a high-resolution technique that can be used stand alone.

Given several prominent successes in high-resolution structures of small structures without phase plates^4^,^18^, it is reasonable to assume that complexes such as hPrx3 could also be solved to high-resolution by a defocus-based approach. However, because of the effort required, this is unlikely to become the norm in the short term.

At the time of writing of this manuscript, according to the Electron Microscopy Data Bank (URL: http://www.emdatabank.org), single-particle cryo-EM has been used to produce 237 near-atomic resolution structures with around 136 structures being either for systems related to ribosomes or highly symmetric viruses. The number of cryo-EM structures solved to 8 Å or higher resolution in 2014 was 185. Of the ∼3,000 total released EM maps, ∼70% have a resolution of 10 Å or lower. Comparison of these numbers with X-ray crystallography depositions^5^ reveals the significant potential to grow the cryo-EM output and increase the number of laboratories participating in such an endeavour.

We believe that by using VPP technology, the potential for the study of biological systems that challenge cryo-EM can be significantly increased, thereby enabling visualization of even smaller protein complexes, highly flexible proteins or systems requiring contrast-reducing solutes. VPP single-particle analysis will enable higher throughput by increasing the success of cryo-EM sessions, which may have previously failed due to ice thickness, poor sample purity or insufficient particle numbers. At the same time, VPP also has the potential to completely replace negative staining, which, despite obvious artefacts in many instances, is still widely used for difficult samples and in the initial screening stages of promising cryo-EM projects that may reach near-atomic resolution.

Taken together, the VPP with its tolerance for recalcitrant specimens may follow the success of the Zernike phase plate in light microscopy and find widespread adoption for routine use in the determination of sub-nanometre resolution structures through single-particle analysis.

## Methods

### Protein expression and purification

Prx3 was purified as described previously^11^. Briefly, protein expression was performed in Rosetta (DE3) cells (Novagen), which were lysed in binding buffer (20 mM HEPES, 150 mM NaCl and 10 mM imidazole, pH 8.0) using a cell press (Microfluidics M110-P lab homogenizer). Soluble fraction were subjected to affinity purification using a 5-ml HisTrap FF column (GE Healthcare and successively subjected to gel filtration using a HiLoad 16/60 Superdex 200 column (GE Healthcare) using imidazole-free gel filtration buffer (20 mM HEPES and 150 mM NaCl, pH 8.0). His-Tag cleavage was mediated by recombinant TEV protease in the presence of 2 mM beta-mercaptoethanol followed by removal of cleaved His-tags and uncleaved protein using a 1-ml HisTrap column (GE Healthcare).

### Preparation of vitrified specimen

For the first data set, frozen-hydrated specimens were prepared on plasma-cleaned holey EM grids (Quantifoil R2/1) using a Vitrobot Mark III (FEI) 4 s blotting time, 85% humidity and −1 mm blotting offset. For the second data set, frozen-hydrated specimens were prepared on plasma-cleaned holey EM grids (Quantifoil R1.2/1.3) using a Vitrobot Mark III (FEI) 4 s blotting time, 85% humidity and −1 mm blotting offset.

### EM and image processing

For the first data set, 44 movies were manually collected on a Titan Krios electron microscope (FEI) operated at 300 kV and equipped with a K2 Summit direct detector, a quantum energy filter (Gatan) and an FEI phase plate (FEI). Movies comprising 10 frames and a total dose of 20 e^−^ per Å^2^ were recorded on a K2 Summit direct detection camera (Gatan) at a calibrated magnification of 37,000 corresponding to a magnified pixel size of 1.35 Å.

The recorded movies showed obvious signs of drift and were subjected to combined global^19^ and local movement correction by optical flow analysis^20^. One micrograph was subjected to e2boxer in EMAN2 (ref. 21) for initial particle picking. The particles were extracted in Relion^22^ using a box size of 152 pixels. This small data set comprising 500 particles was used for reference-free two-dimensional (2D) classification in Relion with 10 classes. Of those 10 classes, three classes were deemed suitable to represent the three dominant views—top view, side view and tilted view. These three classes served as templates for automatic particle picking in Relion yielding 30,000 particles. 2D classification of the full data set was used to remove particles that were either false positives or belonged to classes featuring touching particles resulting in a data set of 10,000 particles. The reduced data set was subjected to three-dimensional (3D) classification using the crystal structure of bovine Prx3 dodecamers as initial model after low-pass filtering it to 60 Å, which produced a featureless toroid. 3D classification was used to group the images into three 3D classes imposing D6 symmetry. 7,000 particles were assigned to the most prominent 3D class, which was further processed with auto-refinement in Relion. This yielded a map, which was sharpened in Relion using a negative B-factor of 800. For resolution determination, the so-called ‘gold-standard' Fourier shell correlation (FSC)=0.143 criterion was used. 3D visualizations of the map and rigid-body dockings were carried out in UCSF Chimera^23^.

For the second data set, 700 micrographs were recorded on a Titan Krios electron microscope operated at 300 kV and equipped with a K2 Summit direct detector, a quantum energy filter (Gatan) and an FEI phase plate (FEI). Automatic data collection was carried out with SerialEM^24^ software. Images were recorded in movie mode comprising 15 frames with a total dose of 20 e^−^ per Å^2^ at a magnification of 28,700 and a pixel size of 1.74 Å. The frames were aligned as described by Li *et al*.^19^. Particles (354,000) were semi-automatically picked using EMAN2 (ref. 21) e2boxer software and extracted in Relion 1.3 with a box size of 152. False positives were removed by three rounds of 2D classification followed by four rounds of 3D classification. 8,580 particles from the best looking 3D class were subjected to 3D auto-refinement in Relion. Particle-based beam-induced motion correction was performed as part of the movie processing. The produced map was then sharpened in Relion with automatically estimated B-factor of −137. The data collection parameters and refinement statistics for the second data set are summarized in Table 1.

## Additional information

**Accession codes:** The cryo-EM map of the 4.4-Å resolution reconstruction has been deposited in the Electron Microscopy Data Bank (EMDB code: EMD-3233). In addition, we provide access to the raw images of hPrx3 recorded with and without the use of VPP (URL: http://dx.doi.org/10.6019/EMPIAR-10050).

**How to cite this article:** Khoshouei, M. *et al*. Volta phase plate cryo-EM of the small protein complex Prx3. *Nat. Commun.* 7:10534 doi: 10.1038/ncomms10534 (2016).

## Supplementary Material

Supplementary Information Supplementary Figures 1-3

## Figures and Tables

**Table 1:** Data collection parameters and refinement statistics.

*Data collection parameters*	
Pixel size (Å)	1.74
Defocus (nm)	20
Voltage (kV)	300
Electron dose (e^−^ per Å^2^)	20
	
*Refinement and modelling statistics*	
Particles for refinement	8,500
Resolution (Å)	4.4
Map sharpening B-factor (Å^2^)	−137
Cross-correlation coefficient for PDB 1ZYE	0.85
Cross-correlation coefficient for PDB 1QMV (dimer)	0.84
