# Supplementary material for: Volta phase plate cryo-EM of the small protein complex Prx3
Source: Nat Commun. 2016 Jan 28;7:10534. doi: 10.1038/ncomms10534 (PMC4738354; doi:10.1038/ncomms10534)
Supplement: Supplementary Information — Supplementary Figures 1-3 [file ncomms10534-s1.pdf]

Supplementary information

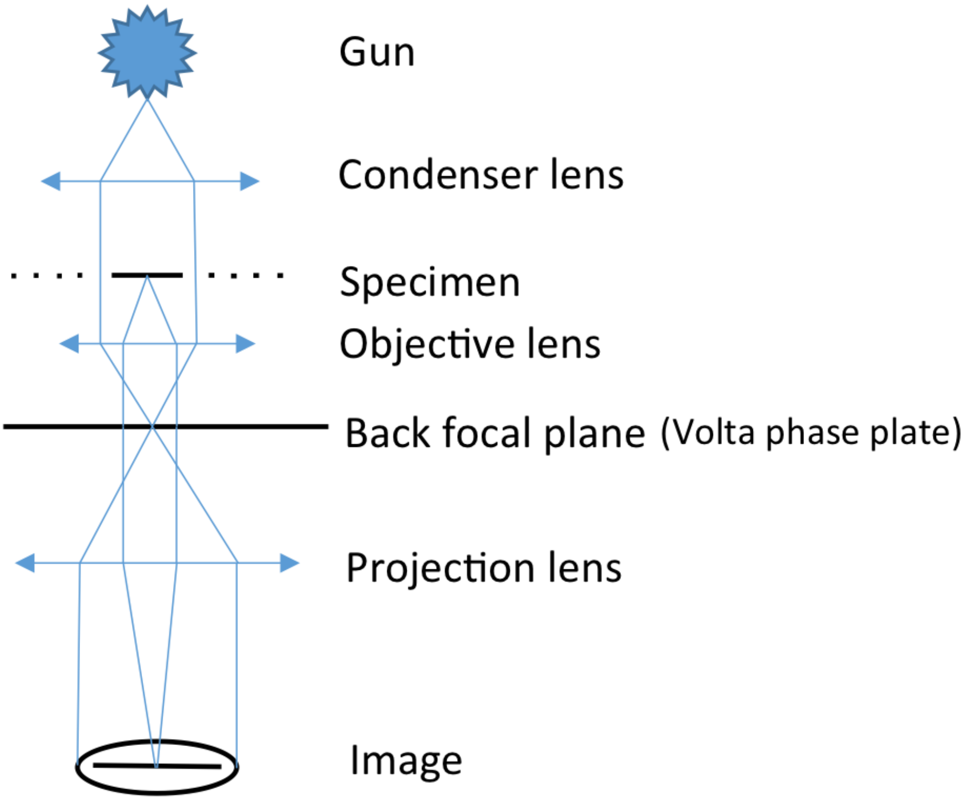

Supplementary Figure 1 – Schematic diagram of VPP. The VPP is a thin carbon film, which can be inserted instead of an objective aperture. Activation by the central diffraction beam in the back focal plane creates a localized phase shift in the centre of the film.

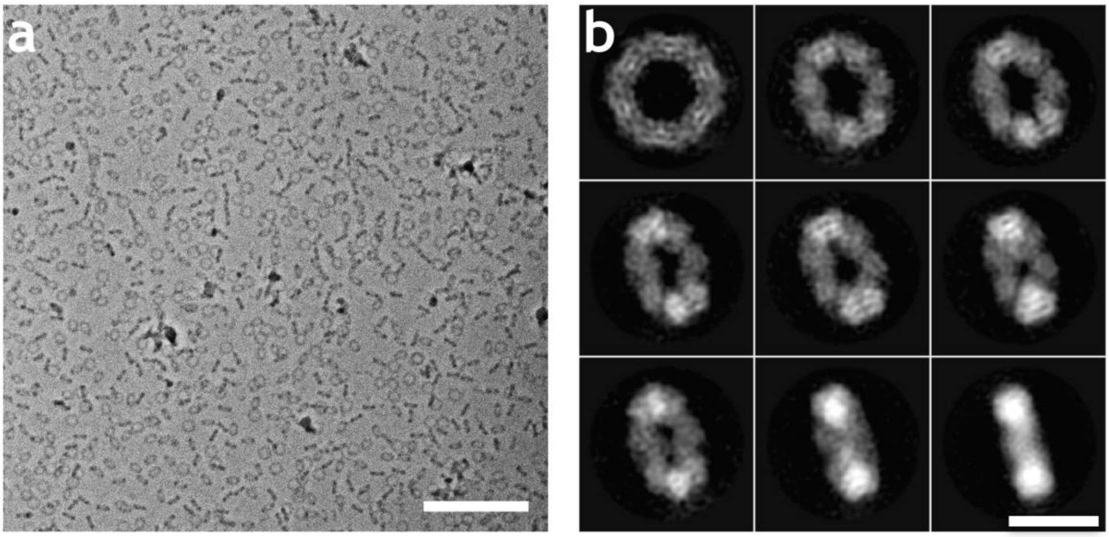

Supplementary Figure 2 – hPrx3 assumes random orientation in thick ice. (**a**) In-focus electron micrograph of hPrx3 particles (Scale bar = 100 nm). (**b**) Nine representative 2D class averages of hPrx3 (Scale bar = 10 nm).

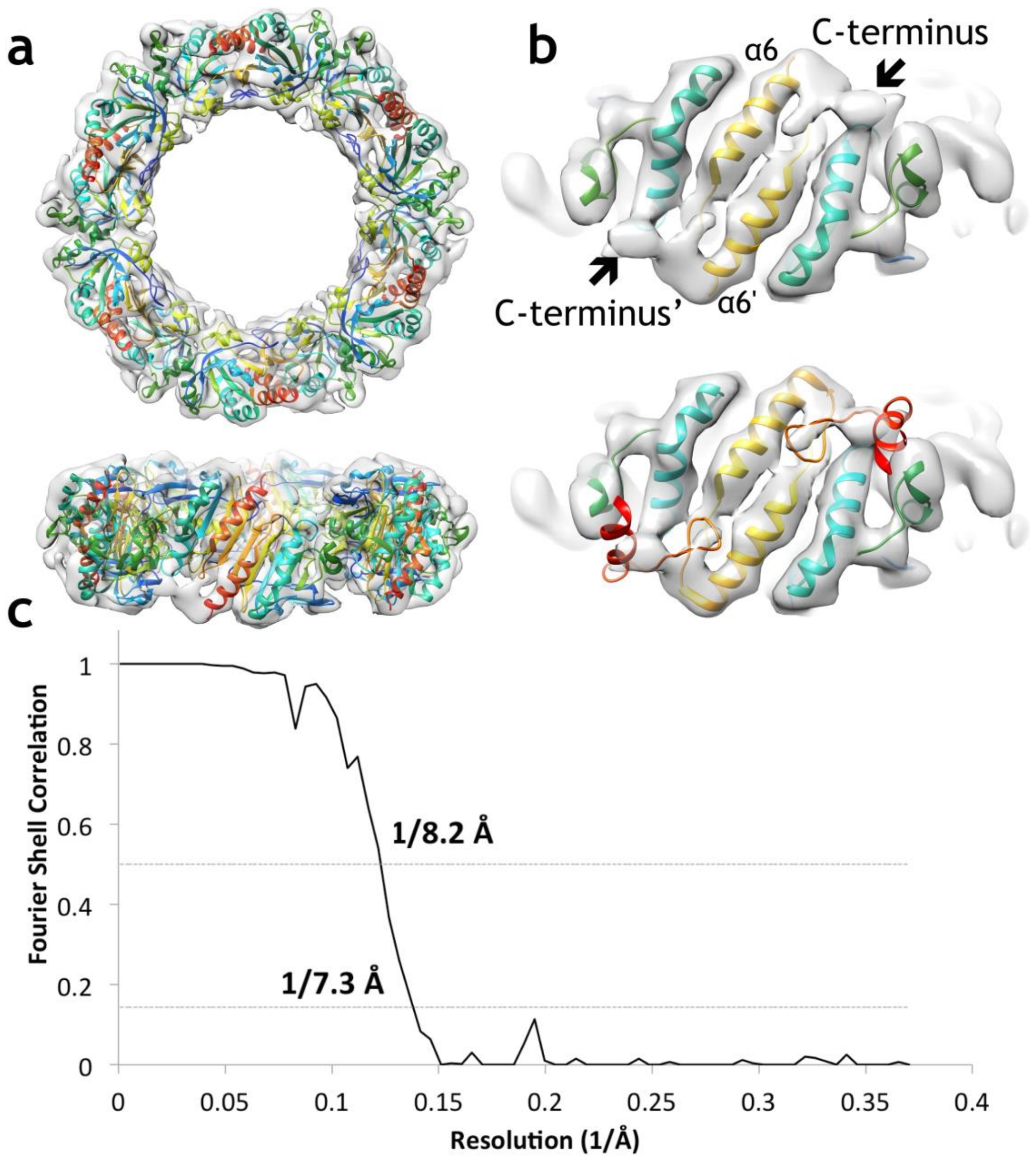

Supplementary Figure 3 – Pseudo-atomic interpretations of hPrx3 reconstructions at 7.3 Å resolution. (**a**) Top and side-view of hPrx3 reconstructions with docked structure of bovine Prx3 (PDB 1ZYE). (**b**) Anti-parallel dimers of hPrx3 feature additional electron density protruding from helix α6 in VPP reconstructions relative to PDB 1ZYE, which can be assigned to a folded C-terminus (red). This feature has been seen for other Prx isoforms such as human Prx2 (PDB 1QMV). (**c**) Fourier-shell correlation of Prx reconstruction shows a resolution of 7.3 Å based on the "gold-standard" FSC=0.143 criterion.
